# A comparative study on the effect of dopamine vs phenylephrine in improving the cutaneous analgesic effect of mexiletine in rats

**DOI:** 10.1186/s13741-023-00314-2

**Published:** 2023-06-13

**Authors:** Kesong Zheng, Mingming Han, Fang Kang, Chengwei Yang, Juan Li

**Affiliations:** Department of Anesthesiology, Division of Life Sciences and Medicine, the First Affiliated Hospital of USTC, University of Science and Technology of China, Hefei, 230036 Anhui China

**Keywords:** Dopamine, Phenylephrine, Mexiletine, Cutaneous analgesia, Sensory blockage, Cutaneous trunci muscle reflex

## Abstract

**Background:**

The present study aimed to compare the effects of the combined administration of two adjuvants, dopamine and phenylephrine, on the cutaneous analgesic effect and duration of mexiletine in rats.

**Methods:**

Nociceptive blockage was evaluated by the inhibition of response to skin pinpricks in rats via the cutaneous trunci muscle reflex (CTMR). After subcutaneous injection, the analgesic activities of mexiletine in the absence and presence of either dopamine or phenylephrine were assessed. Each injection was standardized into 0.6 ml with a mixture of drugs and saline.

**Results:**

Subcutaneous injections of mexiletine successfully induced dose-dependent cutaneous analgesia in rats. The results revealed that rats injected with 1.8 μmol mexiletine exhibited 43.75% blockage (%MPE), while rats injected with 6.0 μmol mexiletine showed 100% blockage. Co-application of mexiletine (1.8 or 6.0 μmol) with dopamine (0.06, 0.60, or 6.00 μmol) elicited full sensory block (%MPE). Sensory blockage ranged from 81.25% to 95.83% in rats injected with mexiletine (1.8 μmol) and phenylephrine (0.0059 or 0.0295 μmol), and complete subcutaneous analgesia was observed in rats injected with mexiletine (1.8 μmol) and a higher concentration of phenylephrine (0.1473 μmol). Furthermore, mexiletine at 6.0 μmol completely blocked nociception when combined with any concentration of phenylephrine, while 0.1473 μmol phenylephrine alone exhibited 35.417% subcutaneous analgesia. The combined application of dopamine (0.06/0.6/6 μmol) and mexiletine (1.8/6 μmol) resulted in increased %MPE, complete block time, full recovery time, and AUCs compared to the combined application of phenylephrine (0.0059 and 0.1473 μmol) and mexiletine (1.8/6 μmol) (*p* < 0.001).

**Conclusion:**

Dopamine is superior to phenylephrine in improving sensory blockage and enhancing the duration of nociceptive blockage by mexiletine.

## Introdution

Mexiletine is a class I antiarrhythmic drug that exerts membrane-stabilizing effects and blocks the conduction of action potentials by inhibiting sodium channels (Dokken and Fairley 2020). Lidocaine, on the other hand, is an amide-type local anesthetic known for its rapid onset, wide dispersion, and strong penetration, making it widely used in local anesthesia (Beaussier et al. 2018). Tzeng et al. conducted a study using a rat subcutaneous infiltration model to evaluate the intensity and duration of cutaneous analgesia provided by mexiletine and compared it with lidocaine (Tzeng et al. 2007). The findings revealed that mexiletine, by blocking sodium channels, induces reversible sensory loss and exhibits a local anesthetic effect on the skin (Tzeng et al. 2007). Moreover, mexiletine outperformed lidocaine in terms of both the local anesthetic effect and the duration of action in cutaneous analgesia (Tzeng et al. 2007). In addition, Vidya et al. reported the efficacy and safety of mexiletine in controlled clinical trials for neuropathic pain (Challapalli et al. 2019). Thus, further investigation into the effect and modality of cutaneous analgesia with mexiletine is warranted to provide clinical evidence supporting its use.

Adjuvants are drugs that work in conjunction with local anesthetics to help increase their analgesic efficacy (Prabhakar et al. 2019; Swain et al. 2017). Adjuvants effectively shorten the onset time of local anesthetics, prolong the block time of sensory and motor nerves, improve the quality of analgesia, and reduce potential drug-related adverse reactions (Swain et al. 2017). Vasoconstrictors are widely used as adjuvants to improve the duration and quality of analgesics by reducing the absorption of analgesics into the bloodstream (Chen et al. 2016). Dopamine and phenylephrine are two types of vasoconstrictor adjuvants that have been shown to be effective in improving the quality and duration of local anesthetics (Chen et al. 2018; Tzeng et al. 2016; Hung et al. 2017; Silva Neto et al. 2020). This article compares the effects of the combined administration of two adjuvants, dopamine and phenylephrine, on the analgesic effect and duration of mexiletine in rats, providing a basis for future clinical use.

## Materials and methods

### Animals

The whole experimental protocol (ref no. STCACUC1902025) was approved by the Institutional Animal Care and Use Committee of the University of Science and Technology of China. All procedures strictly followed the guidelines from the International Association for the Study of Pain. Male Sprague Dawley rats weighing 200 to 250 g were provided by the Anhui Laboratory Animal Center (Hefei, China) and were housed in the animal facility at the Hospital's Laboratory Animal Center. The rats were maintained under a natural light–dark cycle (12-h light/dark cycle, with the light cycle starting at 7:00 AM) at a room temperature of 23 ± 2 °C and a relative humidity of approximately 40–60%. Food and water were provided ad libitum for the rats.

### Materials

Mexiletine hydrochloride and dopamine hydrochloride were purchased from Sigma-Aldrich Chemical Co. (St. Louis, MO, USA) while (R)-Phenylephrine hydrochloride was obtained from Target Molecule Corp. (Boston, MA, USA). All chemicals used in the experiments were freshly prepared and dissolved directly in normal saline.

### Subcutaneous injection and grouping methods

Before experiments, the rats were raised for 7 days to minimize stress and enhance performance. The subcutaneous injection procedure followed the protocols described in previous reports (Tzeng et al. 2017; Chou et al. 2018). Briefly, the hair on the dorsal surface of the rats' thoracolumbar region (10 cm × 6 cm) was shaved using mechanical means prior to the experiments. A 0.6 ml solution containing the specified drugs was then injected directly into conscious rats using 30-gauge needles on the dorsal surface of the thoracolumbar region. Immediately after the injection, a circular bulge (2 cm in diameter) appeared on the skin surface within 1 min of the injection.

Subcutaneous injection of drugs included the following:

1. Different doses of mexiletine (0.6, 1.8, and 6.0 μmol);
2. Combinations of mexiletine (1.8 and 6.0 μmol) with dopamine (0.06, 0.60, and 6.00 μmol);
3. Combinations of mexiletine (1.8 and 6.0 μmol) with phenylephrine (0.0059, 0.0295, 0.1473 μmol).

All injections were standardized into a volume of 6.0 ml with saline.

The dose of dopamine (0.60 μmol) was chosen based on a previous report (Han et al. 2020). From this dose, we increased and decreased it by 10 times, ultimately selecting three doses (0.06, 0.60, 6.00 μmol). In clinical practice, it is recommended to mix phenylephrine with local anesthetics at a ratio of 1:20,000 to achieve potent and prolonged local anesthesia (as stated on the label). In this study, the selected dose of phenylephrine was 1:20,000 (0.1473 μmol), which was then diluted 5 and 25 times consecutively.

### Evaluation of cutaneous analgesia

The degree of analgesia was assessed in experiment 2 using three parameters: %MPE (percent of maximal possible effect), duration of action, and areas under the curve (AUCs) (8 mice per group). Following the method described in a previous study (Chen et al. 2017), the cutaneous analgesic effect was evaluated by measuring the cutaneous trunci muscle reflex (CTMR) response. This response is characterized by the reflex movement of the skin over the back, which is triggered by twitches of the lateral thoracospinal muscle upon local dorsal cutaneous stimulation at specific time points. To induce the CTMR response, we used a Von Frey filament (No. 15; Somedic Sales AB, Stockholm, Sweden) attached to an 18-gauge end-cut needle, applying a noxious stimulus of 19 ± 1 g. Initially, we observed the CTMR response of a mouse to a needle prick on the opposite side of the wheal. Subsequently, six pinholes, with a frequency of 0.5–1 Hz, were applied within the wheal. Finally, we recorded the number of six pinholes to which the animals failed to respond.

Normal reactions to pinpricks outside the wheal and on the contralateral side were initially observed. Later, six pinpricks at frequencies of 0.5–1.0 Hz were applied to six different points inside each wheal during each test. The number of unresponsive pinpricks after the nociceptive stimulus was recorded. The detection was performed at 0, 2, 5, 10, 15, 20, 25, 30, 40, 50, and 60 min after injection, and every 15–30 min thereafter until full recovery. A blinded assay was implemented, where the researcher evaluating the results was unaware of the drug injection to eliminate potential bias. The analgesic effect was quantified by the number of unresponsive pinpricks. Complete unresponsiveness of all six responses was considered a complete nociceptive blockage, referred to as 100% of the possible effect (PE). The maximum effect of each treatment was defined as 100% of the maximal possible effect (MPE), and the full recovery time was measured from subcutaneous injection to complete recovery. Both the full recovery time and %MPE were recorded.

To minimize the number of experimental animals, shaved areas on the back of rats were evenly divided into four non-overlapping sections. Only one treatment was administered per injection site. Each rat received a maximum of four injections, followed by one day of recovery.

### Statistical analysis

The data were expressed as means ± SD. Paired comparisons among groups in %MPE, duration, and AUCs were analyzed using one-way ANOVA followed by Tukey's honestly significant difference (HSD) test. The AUCs of nociceptive blockage were calculated using GraphPad Prism 7 (GraphPad Software Inc., California, USA). All statistical analyses were performed using SPSS (version 17.0, SPSS Inc., Chicago, IL, USA), and a *p*-value < 0.05 was considered significant.

## Results

**Fig. 1:**
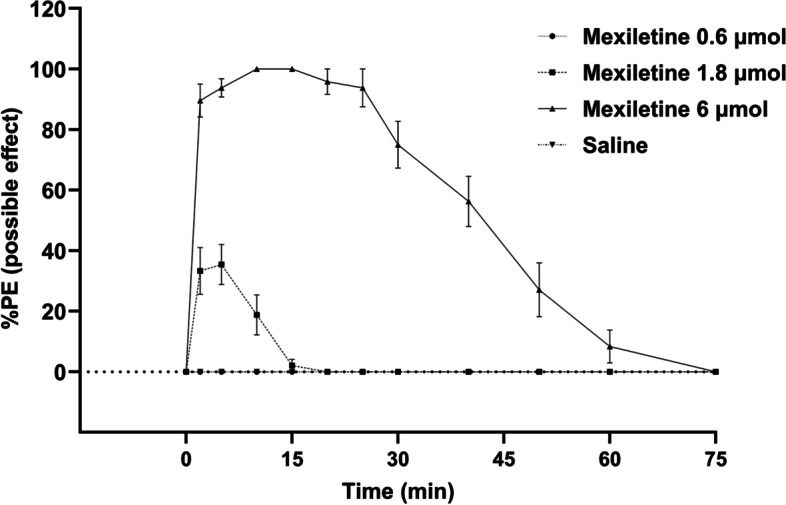
Time courses of cutaneous analgesia after treatments of three doses of mexiletine and saline control. Data are presented as mean ± SD; *n* = 8 rats for each drug dose

Subcutaneous injections of mexiletine successfully induced dose-dependent cutaneous analgesia in rats (Fig. 1). The results revealed that mice injected with 1.8 μmol mexiletine exhibited 43.75 ± 7.00% blockage of pain response (%MPE), while those injected with 6.0 μmol mexiletine showed 100% blockage. Based on the experiments shown in Fig. 1, 0.6 µmol of mexiletine did not produce any analgesic effect, whereas 1.8 µmol and 6 µmol doses demonstrated significant analgesic effects upon subcutaneous injection. Notably, the 6 µmol dose of mexiletine exhibited superior analgesic effects. Furthermore, no significant side effects were observed for any of the mexiletine doses used (data not shown). Consequently, two doses, 1.8 µmol and 6 µmol, were selected for subsequent experiments.

**Fig. 2:**
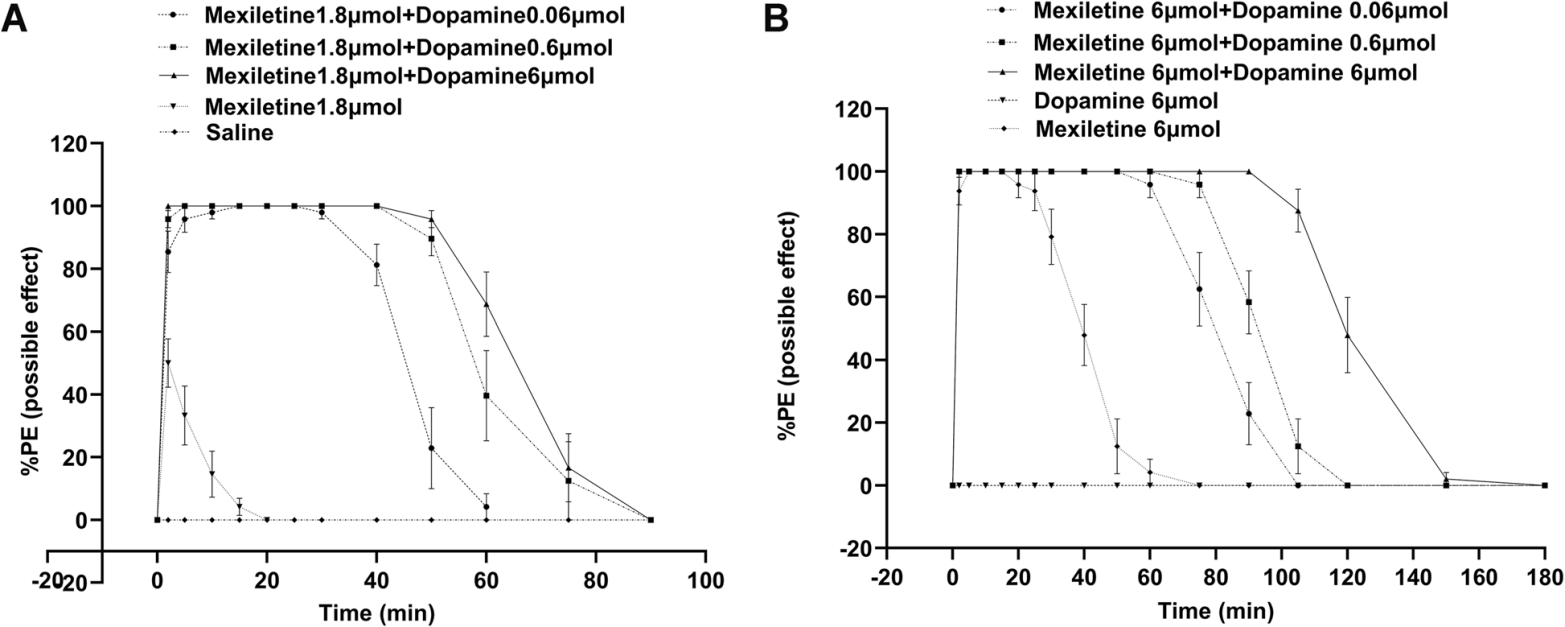
Effects of cutaneous analgesia were seen after the addition of dopamine (0.06, 0.60, or 6.00 μmol) with mexiletine at the concentrations of 1.8 μmol (**A**) or 6.0 μmol (**B**). Data are presented as mean ± SD; *n* = 8 rats for each drug dose

The co-application of mexiletine (1.8 or 6.0 μmol) with dopamine (either 0.06, 0.60 or 6.00 μmol) elicited a full sensory block (%MPE) (Figs. 2A and B). In contrast, the administration of vehicle (saline only) or dopamine alone (6.00 μmol) did not produce any cutaneous analgesic effect (Figs. 2A and B).

**Fig. 3:**
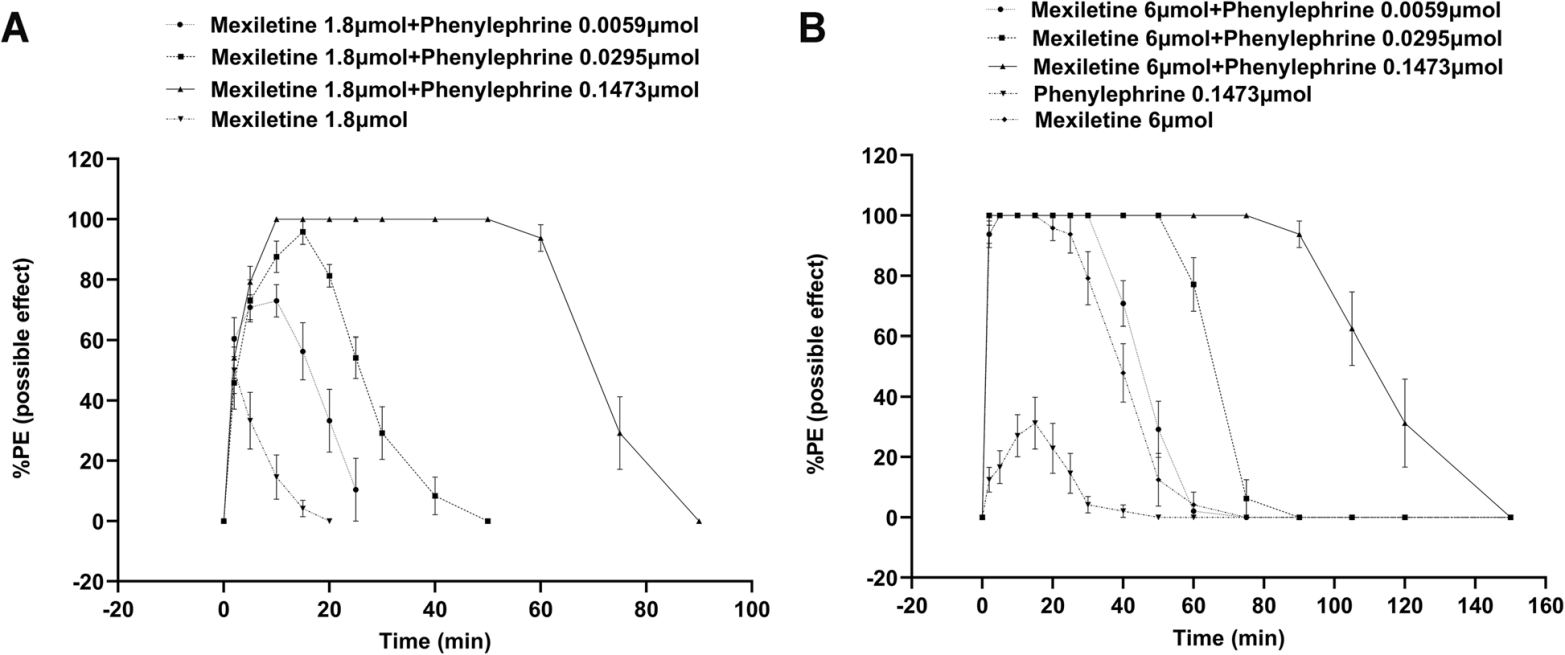
Effects of cutaneous analgesia were seen after the addition of phenelephrine (0.0059, 0.0295, or 0.1473 μmol) with mexiletine at the concentrations of 1.8 μmol (**A**) or 6.0 μmol (**B**). Data are presented as mean ± SD; *n* = 8 rats for each drug dose

The sensory blockage reached 81.25 ± 3.78% and 95.83 ± 4.17% in rats injected with mexiletine (1.8 μmol) and phenylephrine (0.0059 and 0.0295 μmol, respectively). However, full subcutaneous analgesia was observed in rats injected with mexiletine (1.8 μmol) and a higher concentration of phenylephrine (0.1473 μmol), as shown in Fig. 3A. Furthermore, mexiletine at 6.0 μmol completely blocked nociception regardless of the combination with phenylephrine, while 0.1473 μmol phenylephrine alone exhibited 35.417 ± 8.59% subcutaneous analgesia (Fig. 3B). Notably, the application of the saline vehicle did not show any cutaneous analgesic effect.

Tables 1 and 2 present the %MPE, duration and AUCs of rats after the application of each drug alone or in combination. Comparing the application of mexiletine alone (1.8 μmol) with the co-application of either dopamine or phenylephrine, a significant potentiation and prolongation of the block effect of skin nociception were observed. Similarly, comparing the application of mexiletine alone (6.0 μmol) with the co-application of either dopamine or phenylephrine, a significant extension in the duration of cutaneous analgesia was observed as well.

**Table 1:** The percentages of maximum possible effect (%MPE), duration, and the area under the curves (AUCs) of mexiletine alone or co-administration of low dose mexiletine with dopamine or phenylephrine

	%MPE	Duration (min)		AUCs (%MPE × min)
		Complete block time	Full recovery time	
Mex 1.8 μmol	43.75 ± 7.00	0	13.75 ± 1.25	329.163 ± 69.53
Mex 1.8 μmol + Phe 0.0059 μmol	81.25 ± 3.78^a^	0	25.000 ± 0.94	1299.000 ± 136.66^b^
Mex 1.8 μmol + Phe 0.0295 μmol	95.83 ± 4.17^ac^	3.750 ± 1.25	40.00 ± 2.67^ad^	2211.875 ± 198.80^ae^
Mex 1.8 μmol + Phe 0.1473 μmol	100 ± 0^ad^	48.75 ± 1.57^acf^	82.500 ± 2.83^adf^	6811.375 ± 200.12^af^
Mex 1.8 μmol + Dop 0.06 μmol	100 ± 0^ad^	29.625 ± 3.56^acfh^	55.625 ± 3.20^acgh^	4414.75 ± 238.21^acfh^
Mex 1.8 μmol + Dop 0.60 μmol	100 ± 0^ad^	46.625 ± 4.07^acfj^	71.25 ± 3.75^acfk^	5780.25 ± 223.72^acfij^
Mex 1.8 μmol + Dop 6.00 μmol	100 ± 0^ad^	48.000 ± 2.67^acfj^	78.75 ± 62.45^acfj^	6467.750 ± 269.12^acfj^

Data are expressed as mean ± SD (*n* = 8 in each group). Compared with Mex 1.8 μmol, ^a^*p* < 0.001, ^b^*p* < 0.05; compared with Mex 1.8 μmol + Phe 0.0059 μmol, ^c^*p* < 0.001, ^d^*p* < 0.01, ^e^*p* < 0.05; compared with Mex 1.8 μmol + Phe 0.0295 μmol, ^f^*p* < 0.001, ^g^*p* < 0.01; compared with Mex 1.8 μmol + Phe 0.1473 μmol, ^h^*p* < 0.001, ^i^*p* < 0.05; compared with Mex 1.8 μmol + Dop 0.06 μmol, ^j^*p* < 0.001, ^k^*p* < 0.01. The data among the groups were analyzed using one-way ANOVA followed by Tukey’s honest significant difference (HSD) test for paired comparisons. *Mex* Mexiletine, *Dop* Dopamine, *Phe* Phenylephrine

**Table 2:** The percent of maximum possible effect (%MPE), duration, and the area under the curves (AUCs) of mexiletine alone or co-administration of high dose mexiletine with dopamine or phenylephrine

	%MPE	Duration (min)		AUCs (%MPE × min)
		Complete block time	Full recovery time	
Mex 6.0 μmol	100 ± 0	20.625 ± 2.65	61.875 ± 4.11	4046.75 ± 285.99
Mex 6.0 μmol + Phe 0.0059 μmol	100 ± 0	28.125 ± 1.51	58.125 ± 2.98	4410.500 ± 170.70
Mex 6.0 μmol + Phe 0.0295 μmol	100 ± 0	51.75 ± 1.83^ac^	76.875 ± 1.88^bd^	6457.375 ± 169.59^ae^
Mex 6.0 μmol + Phe 0.1473 μmol	100 ± 0	91.375 ± 5.48^acf^	135 ± 5.67^acf^	11,196.875 ± 522.35^acf^
Mex 6.0 μmol + Dop 0.06 μmol	100 ± 0	60.25 ± 2.84^ach^	62.500 ± 2.99^ h^	7879.125 ± 341.36^ach^
Mex 6.0 μmol + Dop 0.60 μmol	100 ± 0	70.25 ± 1.75^acgh^	73.125 ± 1.88^eh^	9150.000 ± 287.38^acfi^
Mex 6.0 μmol + Dop 6.00 μmol	100 ± 0	97.375 ± 2.74^acfjk^	99.375 ± 2.74^acfhjk^	12,103.123 ± 394.53^acfjk^
Saline	0	0	0	0
Dop 6.00 μmol	0	0	0	0
Phe 0.1473 μmol	35.417 ± 8.59	0	27.50 ± 5.18	629.20 ± 194.55

Data are expressed as mean ± SD (*n* = 8 in each group). Compared with Mex 6.0 μmol, ^a^*p* < 0.001, ^b^*p* < 0.05; compared with Mex 6.0 μmol + Phe 0.0059 μmol, ^c^*p* < 0.001, ^d^*p* < 0.01, ^e^*p* < 0.05; compared with Mex 6.0 μmol + Phe 0.0295 μmol, ^f^*p* < 0.001, ^g^*p* < 0.01; compared with Mex 6.0 μmol + Phe 0.1473 μmol, ^h^*p* < 0.001, ^i^*p* < 0.01; compared with Mex 6.0 μmol + Dop 0.06 μmol, ^j^*p* < 0.001; compared with Mex 6.0 μmol + Dop 0.60 μmol, ^k^*p* < 0.001. The data among the groups were analyzed using one-way ANOVA followed by Tukey’s honest significant difference (HSD) test for paired comparisons. *Mex* Mexiletine, *Dop* Dopamine, *Phe* Phenylephrine

Co-application of 1.8 μmol mexiletine and 0.06 μmol dopamine significantly increased %MPE, complete block time, full recovery time, and AUCs compared to rats treated with mexiletine 1.8 μmol and either 0.0059 μmol or 0.1473 μmol phenylephrine (*p* < 0.001). Moreover, the co-administration of 1.8 μmol mexiletine and 6.00 μmol dopamine resulted in increased %MPE, complete block time, full recovery time, and AUCs compared to the co-administration of mexiletine 1.8 μmol and 0.1473 μmol phenylephrine (*p* < 0.001). Furthermore, when compared to rats treated with mexiletine (6.0 μmol) and phenylephrine (0.0059 μmol), an increase in the complete block time and AUCs was observed in rats injected with 6.0 μmol mexiletine and 0.06 μmol dopamine (*p* < 0.001). Additionally, compared to rats injected with mexiletine (6.0 μmol) and phenylephrine (0.0295 μmol), the complete block time and AUCs increased in rats treated with 1.8 μmol mexiletine and 0.60 μmol dopamine (*p* < 0.001). Lastly, when compared to rats injected with mexiletine (6.0 μmol) and phenylephrine (0.1473 μmol), the full recovery time decreased in rats treated with 6.0 μmol mexiletine and 6.00 μmol dopamine (*p* < 0.001).

Moreover, intraperitoneal administration of mexiletine at 6.0 μmol, dopamine at 6.00 μmol, phenylephrine at 0.1473 μmol, or any combination of these compounds failed to induce cutaneous analgesia. Additionally, all treated rats completely recovered from the injections afterwards.

## Disscusion

In agreement with the previous study (Ning et al. 2017), the current research indicated that mexiletine injection could elicit subcutaneous analgesia through local infiltration in a dose-dependent manner. More importantly, the results from this study showed that the application of both dopamine and phenylephrine improved the sensory blockage and enhanced the duration of the nociceptive block caused by mexiletine, with dopamine being superior to phenylephrine.

All doses of dopamine and phenylephrine enhanced sensory blockage and prolonged the duration of nociceptive blockage by mexiletine. Relevant studies have shown that the combined application of dopamine or phenylephrine can increase the analgesic effect of local anesthetics (Chen et al. 2018; Tzeng et al. 2016; Hung et al. 2017; Holmberg et al. 2019). It has been suggested that local anesthetics suppress neural impulses by inhibiting sodium currents in the nerves (Tikhonov and Zhorov 2017). Consistently, lidocaine and its analog mexiletine have been shown to be sodium channel blockers (Otuki et al. 2017). In a previous study, lidocaine produced dose-dependent analgesia in rats (Chen et al. 2016). Additionally, spinal blockage can also be induced by intrathecal application of mexiletine in rats (Chen et al. 2012).

Postoperative pain imposes both physical and psychological burdens on patients and can lead to abnormalities in gastrointestinal function, cardiopulmonary function, coagulation function, endocrine metabolism, and other complications, seriously affecting patient recovery. Effective postoperative analgesia not only alleviates pain and improves patient satisfaction but also reduces postoperative complications, shortens hospital stays, and promotes rapid recovery. Therefore, postoperative analgesia that can effectively relieve pain has become a crucial aspect of enhancing recovery after surgery. Cutaneous analgesia achieved through the application of local anesthetic drugs is considered an acceptable method for pain management due to its lower incidence of side effects (Chiu et al. 2019). The duration of blockage serves as an important indicator in clinical practice, and its prolongation represents a significant goal in postoperative pain therapy (Marhofer and Brummett 2016). Early pain management trials involved widespread use of opioids, such as morphine and its derivatives, fentanyl, sufentanil, buprenorphine, tramadol, and others. Although these approaches yielded reasonable success, opioids were often associated with systemic complications, including respiratory depression, nausea, vomiting, and pruritus. Consequently, there has been an increasing utilization of adjuncts (such as opioids, adrenaline, α2-adrenoreceptor agonists, steroids, and other anti-inflammatory agents) in combination with local anesthetics to enhance the quality of peripheral nerve blocks (Swain et al. 2017).

Adrenaline is a vasoconstrictor traditionally used as an adjuvant to improve the quality and duration of analgesia (Holmberg et al. 2019), as it has been previously shown to reduce the diffusion of local anesthetics into the bloodstream (Sheikh et al. 2017; Wiesmann et al. 2018). Interestingly, in the current study, subcutaneous phenylephrine at a dose of 0.1473 μmol resulted in a 35.417% blockage (%MPE), which is in line with previous reports suggesting that phenylephrine itself can induce cutaneous anesthesia through the activation of various subtypes of α1-adrenoceptors (Drummond et al. 2018). The results of this study indicate that the difference in analgesic effect caused by the synergistic use of dopamine and phenylephrine with mexiletine is primarily dependent on the doses of dopamine and phenylephrine. Phenylephrine primarily stimulates α receptors, with a much stronger effect on α1 receptors than on α2 receptors. Dopamine, on the other hand, is a sympathomimetic vasoactive drug that stimulates dopamine receptors (DA1, DA2), β1 receptors, and α receptors depending on the dose, and also promotes the release of norepinephrine. The vasoconstrictive effect of dopamine is stronger than that of phenylephrine, resulting in a stronger synergistic analgesic effect. However, dopamine has a shorter half-life (10 min) compared to phenylephrine (60 min), resulting in a shorter duration of analgesia. It is important to note that in this study, dopamine and phenylephrine were injected subcutaneously and did not cross the blood–brain barrier in mice. Therefore, the conclusions of this study may not be relevant to the central nervous system and the activation of dopaminergic and adrenergic pathways. Thus, we speculate that the differences observed between dopamine and phenylephrine as adjuvants in combination with mexiletine, as found in this study, are mainly due to their dose-related vasoconstrictor effects, which require further experimental verification.

In conclusion, subcutaneous application of mexiletine produced dose-dependent cutaneous analgesia. Both dopamine and phenylephrine improved sensory blockage and enhanced the duration of nociceptive blockage by mexiletine, but dopamine had a superior effect compared to phenylephrine.

## Data Availability

The datasets used and/or analysed during the current study are available from the corresponding author on reasonable request.

## Abbreviations

AUCs: Areas under the curve
PE: Possible effect
MPE: Maximal possible effect
HSD: Honestly significant difference
