# Correction: A comparative study on the effect of dopamine vs phenylephrine in improving the cutaneous analgesic effect of mexiletine in rats

**DOI:** 10.1186/s13741-024-00361-3

**Published:** 2024-01-11

**Authors:** Kesong Zheng, Mingming Han, Fang Kang, Chengwei Yang, Juan Li

**Affiliations:** Department of Anesthesiology, the First Affiliated Hospital of USTC, Division of Life Sciences and Medicine, University of Science and Technology of China, Hefei, 230001 Anhui China

**Correction: Perioperative Medicine 12, 26 (2023)**

**https://doi.org/10.1186/s13741-023-00314-2**

Following publication of the original article (Zheng et al. 2023), the author would like to reorder details in affiliation 1. The correction details for the affiliation 1 are: Department of Anesthesiology, the First Affiliated Hospital of USTC, Division of Life Sciences and Medicine, University of Science and Technology of China, Hefei, 230001, Anhui, China.

## References

[CR1] Zheng K, Han M, Kang F (2023). A comparative study on the effect of dopamine vs phenylephrine in improving the cutaneous analgesic effect of mexiletine in rats. Perioperative Medicine.

